# May I see your ID, please? An explorative study of the professional identity of undergraduate medical education leaders

**DOI:** 10.1186/s12909-017-0860-0

**Published:** 2017-02-01

**Authors:** Kristina Sundberg, Anna Josephson, Scott Reeves, Jonas Nordquist

**Affiliations:** 1 Medical Case Centre, Department of Medicine (Huddinge), Karolinska Institutet, M54 SE-141 86 Stockholm, Sweden; 2 Department of Neuroscience, Karolinska Institutet, Stockholm, Sweden; 3 Faculty of Health, Social Care & Education, Kingston University & St George’s, University of London, London, United Kingdom

## Abstract

**Background:**

The mission of undergraduate medical education leaders is to strive towards the enhancement of quality of medical education and health care. The aim of this qualitative study is, with the help of critical perspectives, to contribute to the research area of undergraduate medical education leaders and their identity formation; how can the identity of undergraduate medical education leaders be defined and further explored from a power perspective?

**Methods:**

In this explorative study, 14 educational leaders at a medical programme in Scandinavia were interviewed through semi-structured interviews. The data was analysed through Moustakas’ structured, phenomenological analysis approach and then pattern matched with Gee’s power-based identity model.

**Results:**

Educational leaders identify themselves more as mediators than leaders and do not feel to any larger extent that their professional identity is authorised by the university. These factors potentially create difficulties when trying to communicate with medical teachers, often also with a weaker sense of professional identity, about medical education.

**Conclusions:**

The perceptions of the professional identity of undergraduate medical education leaders provide us with important notions on the complexities on executing their important mission to develop medical education: their perceptions of ambiguity towards the process of trying to lead teachers toward educational development and a perceived lack of authorisation of their work from the university level. These are important flaws to observe and correct when improving the context in which undergraduate medical education leaders are trying to develop and improve undergraduate medical programmes. A practical outcome of the results of this study is the facilitation of design of faculty development programmes for educational leaders in undergraduate medial education.

**Electronic supplementary material:**

The online version of this article (doi:10.1186/s12909-017-0860-0) contains supplementary material, which is available to authorized users.

## Background

The field of medicine has been referred to as the very embodiment of the concept of professional identity [1]; to be a physician is one of the most explicit examples, both historically and currently, of a professional identity. Identity may be defined in several different ways but is defined by Gee [2] as the way individuals understand themselves, interpret experiences, present themselves and wish to be perceived by others as well as how they are recognized by the broader community [2]. The context of medical education is first and foremost an arena for knowledge development amongst medical students, but also an arena for the development of their professional identity as physicians [3–5]. The importance of researching and exploring these processes for development of medical students’ professional identity has been stressed by Monrouxe [3] because of its importance in a wider perspective: how medical students perceive and construct their professional identity affects their well-being as well as their relationship with patients and co-workers. Professional identity develops through the process of secondary socialisation where a common body of professional knowledge is internalised by the new member of the profession [6]. However, it is not solely medical students who on the undergraduate medical education arena go through the secondary socialisation processes towards the formation of a professional identity.

Within the realm of undergraduate medical education, the literature also highlights the often complex and cumbersome identity formation of medical educators [1, 7–10]. Stenfors-Hayes et al. [7] explores how medical educators lack interpersonal motives for professional development which in turn can be derived from factors such as low status of teaching, competing identities (such as the roles of being a clinician and/or a researcher) as well as a lack of support, influence and responsibility within the teaching role. Taylor et al. [8] have also identified medical educators teaching in clinical settings as identifying themselves first and foremost as clinicians and physicians and only secondary as teachers. Stone et al. [1] have highlighted how medical educators believe that the role as a physician and the role as a teacher have several characteristics in common, even though the overlap has not been clearly defined. As teachers in medical education they also expressed a lack of a community of peers to discuss educational issues with [1]. Bartle & Thistlethwaite [9] describe how the success of clinical educators is measured in terms of research productivity and clinical work instead of teaching; they identify themselves primarily as clinicians and the identity as an educator is only seen as secondary. Kumar et al. [10] even points out how clinicians who favour teaching over research may experience feelings of marginalisation and inauthenticity. All in all, the professional identity and identity creating processes of teachers within medical education is often both challenging and complex.

However, apart from students and teachers the undergraduate medical education arena also contains other influential players such as deans, program directors and course leaders: medical education leaders. They are key figures in the process of steering educational reform towards 21st century medical education and as a result, reform of health systems [11]. Their mission is to strive towards the enhancement of quality of medical education and health care worldwide [12]. More specifically, their task is to develop the quality of the curricula through initiating, implementing and evaluating educational reforms [13]. However, research on the identity and identity formation of undergraduate medical education leaders is very limited. Of the few undertaken, Lieff et al. [14] explored the notion of “academic identity” among undergraduate medical education leaders; a professional identity connected primarily to medical education. The study was conducted in connection to a faculty development programme and factors from three domains appeared to contribute to the formation of this identity: the personal domain (cognitive and emotional factors unique to each individual), the relational domain (connections and interactions with others) and the contextual domain (faculty development and external work environment) [14]. Sundberg et al. [15] recognised identity as a key feature when undergraduate medical education leaders are trying to lead teachers along in educational development processes while aspects of identity formation of medical education leaders and the importance of their communities of practice is explored by Bolander Laksov & Tomson [16]. Hence, professional identity formation is related to at least three different groups within the realm of undergraduate medical education: students, teachers and educational leaders.

While identity formation is an important and central process in the undergraduate medical education arena, the literature points toward the fact that critical perspectives are rarely used in the research area of medical education leadership. Critical perspectives can offer and contribute with insights on how leadership can function as a process between all involved stakeholders instead on only focusing on the traditional power structure between leaders and followers: being in charge and following the person in charge [17, 18]. Hence, the aim of this study is to contribute to the currently limited research on medical education leaders and their identity formation; specifically to shed light on the identity of undergraduate medical education leaders. Given the lack of critical perspectives the study will focus on the concept of identity development for medical education leaders through addressing the following research question: how can the identity of undergraduate medical education leaders be defined and further explored from a power perspective?

## Methods

### Context

The context of the study was an undergraduate medical programme, with a duration of five and a half years. The programme was situated at a medical university in Scandinavia which at the time of the interviews was admitting approximately 240 students every year and having a curriculum with an integrative and thematic character. Within the undergraduate medical programme there were teachers who had been appointed the responsibility of leading fellow teachers and/or supervisors in the process of implementing the curriculum. Their task as undergraduate medical education leaders was to keep the process of development and educational change alive.

### Design and theoretical proposition

This qualitative study adopts a phenomenological approach; a qualitative methodology which aims to understand social phenomena from the perspectives of those who have experienced it [19]. The phenomenological approach aims to elicit the essence of individuals’ lived experiences [20]. Individual’s experiences are generated through in-depth interviews which explores different components of social phenomena, significant to the specific individuals [21]. Given the lack of critical perspectives in the medical education leadership literature, though well used in the social sciences, the interview guide was developed around the two sensitizing concepts of *power* and *resistance* in relationship to the experience of being an undergraduate medical education leader and leading change. Sensitizing concepts are often used in social sciences to draw attention to important features of social interaction [22] as well as background ideas that inform the overall research problem [23]. The study represents a sub-set of data from a larger PhD research project exploring the notion of power and resistance in connection to educational leadership, both in undergraduate medical education and nursing education.

The theoretical proposition chosen to highlight the results of this study is Gee’s [2] definition of an educational leader as an “institution-identity” (“i-identity”), derived from his power-based identity model. Other examples of thematic identities included in the model are nature-identities (e.g. being an identical twin – a state of nature), discourse-identities (e.g. being charismatic – an individual trait shaped in dialogue with other people) and affinity-identities (e.g. being a Star Trek fan – sharing experiences of specific practices) [2] (see Table 1).

**Table 1:** Gee’s power-based identity model: “Four ways to view identity” (2001)^a^

Process		Power	Sources of power
Nature-identity: a state	*developed from*	forces	in nature
Institution-identity: a position	*authorized by*	authorities	within institutions
Discourse –identity: an individual trait	*recognized in*	the discourse/dialogue	of/with “rational” individuals
Affinity-identity: experiences	*shared in*	the practice	of “affinity groups”

^a^Table reproduced with permission of JP Gee from his original work “Identity as an analytical lens for research in education” (2001)

According to Gee [2] the perspective of the identity as an education leader is an institutional one. It is considered a *position* at an institution since it is not an identity that was given by nature or that could have been created by the person holding the position him- or herself. The source of *power* that determines the position/i-identity is a set of authorities. In turn, the *source of the power* stems from within an institution, here a medical university, and the particular process through which this kind of power works is through *authorisation*. The position is being authorised by the university including granting the rights and responsibilities that goes with the position. However, i-identities may be put on a continuum depending on how actively or passively the person fulfils his or her duties in connection to the position. Also, this given identity may either be sustained or ignored by the university depending on if certain kinds of interactions and dialogues take place often enough [2]. The power-based identity model was chosen as it includes the identity of educational leaders, the i-identity, and aligns well with the applied power perspective.

### Data collection

The data collection was conducted through semi-structured interviews during December 2011 – April 2012 by the first author [Additional file 1]. 26 educational leaders were identified in the undergraduate medical programme via their website. Out of 23 invited leaders 16 accepted to be interviewed, one declined and eight did not reply to the request. The 14 informants purposefully sampled and included in this study were identified as being medical educational leaders on one out of three leadership levels [24]: line level e.g. theme leaders and course leaders. The main task of the informants as medical education leaders were to implement the curriculum for the undergraduate medical programme and hence to encourage and lead teachers and/or supervisors within this process. However, the majority of the informants also functioned as teachers, clinicians, supervisors and/or researchers alongside their duties as medical education leaders. Out of the 14 informants four were women. Regarding academic qualifications 13 of the informants were associate professors while one was a professor and regarding professional status 12 were physicians out of which 10 were chief physicians.

### Data analysis

The interviews were transcribed verbatim and the data analysis was conducted in the following two steps:

1. Data analysis guided by Moustakas’ structured, phenomenological analysis approach [21, 25]
2. Pattern matching using Gee’s theoretical proposition of the i-identity [2]

#### Step 1

Moustakas’ structured phenomenological analysis approach is a qualitative instrument for data analysis derived from transcendental phenomenology [26]. This branch of phenomenology is based on the principles identified by Husserl and provides an alternative to for example hermeneutics [27]. The analysis process starts by developing a list of significant statements about how the informants have experienced a specific phenomenon i.e. their identity as educational leaders. This process is referred to as horizontalization of the data. The data is then clustered into larger themes and in the final stage the researcher writes a description of the phenomenon including both *what* the informants have experienced of the phenomenon (textual description) and *how* the experience happened (structural description) [25].

#### Step 2

To boost the level of transferability of the results of this study, an analytical procedure called “pattern matching” was conducted as step two of the data analysis. It is a strategy for aligning data to theoretical propositions; trying to link the empirical pattern with a theoretical pattern and also to explain why certain components may not match [26]. Pattern matching using Gee’s [2] theory of the i-identity was performed. Both the theoretical patterns and the empirically found patterns will be presented below with the help of three headings: *perceived identity*, *identity process* and *power and identity*. The headings have been derived by the authors from Gee’s [2] definition of identity used in this study as well as his power-based identity model [2] used for the pattern matching. The empirically found patterns will be exemplified through a textual and structural description including quotes from the informants. Comparisons between the theoretical and empirical patterns will be found in the discussion.

### Trustworthiness

The trustworthiness of the study is enhanced through a number of ways during the research process. Dependability is created through agreement on themes as well as the pattern matching process conducted within the research team during the analysis process. The usage of the pattern matching procedure in the analysis stage and hence potential theoretical transference also heightens the level of transferability of the result [20]. Through the theoretically connected sensitizing concepts of *power* and *resistance*, theory also frames the research question. This potentially ensures methodological congruence [28, 29].

## Results

### Perceived identity

A strong feeling in connection to the perceived identity was ambiguity. Even though agreeing on trying to lead teachers towards educational goals, often through mediation, feelings of ambivalence towards the identity label “leader” were expressed by the informants. The ambivalence was most clearly expresses by the course leaders, having the closest contact with teachers and at the same time the strongest feelings of having difficulties leading in a traditional sense. The feeling of ambiguity sometimes also was described as a result of holding an educational leadership position but at the same time being active as a researcher and/or a clinician:

*So, I haven’t really thought of it as a…as a managing role. Or I have hardly thought of it as a leadership role but instead… I am a course leader but… I design and execute the course after consultation and in cooperation with the other teachers.*

(Participant no 6)

*And then you try to do some clinical work…your everyday work. And then you try to do some research. So it’s…it’s an impossible equation, really. So, now I have become more and more engaged in education and then the other things have to be put aside.*

(Participant no 9)

Turning away from the perceived identity of being a traditional leader the informants first and foremost identified themselves as a person who is a negotiator and a diplomat trying to coordinate, negotiate and communicate between different interests and tribes: different departments, different specialties, different hospital sites, different learning cultures, clinical work and university/education, preclinical and clinical, research and education, hospitals and primary care and also across courses and themes. Being and acting as a mediator was felt to be necessary to get the courses, themes and programme to run accordingly and was therefore also perceived as an important and central task that strongly influenced the identity. It was also described as a task that was challenging to overcome.

*I think it was important not to confront too much but instead…in a dialogue agree on that this is the way it could work, this is the way it could turn out successfully. Diplomacy.*

(Participant no 6)

Also in connection to the idea of mediating was the perception of neutrality. Viewing oneself as neutral or originating from an alternative sphere was a strong identity factor among some of the informants. They expressed that this aspect of the identity made it possible for them to mediate and negotiate more successfully between groups in development processes.

*I had stayed away. I wasn’t stigmatized at any department and everybody viewed me as pretty harmless and hopefully a nice person.*

(Participant no 12)

*Well, I guess it was something of a compromise and I was a neutral enough person among the other departments to an acceptable degree.*

(Participant no 2)

### Identity process

Being an educational leader in undergraduate medical education was described first and foremost as both being perceived as and feeling unofficial. These perceptions were connected to difficulties and struggles getting teachers and supervisors engaged in educational change and development. This was in turn perceived to be caused by conflicts of interest; differing visions shared with the hospitals and health care production as well as a lack of influence over teachers with expert status, at times employed at other departments.

*Well… I’m not a… I can’t formulate it like a head of clinic: “you will do this now”. Instead, I can just plea to them: “if we do it like this it will work out well”. And then, being one of these unofficial leaders for a course or how to put it, I have made sure that we have days where we can plan the course.*

(Participant no 9)

*Being a course leader is a pretty fuzzy…well… its’ not as formalised I would say. Well…of course you have a mission to perform but… You’re not a line manager in the same way as a head of clinic, so you’re not a manager…or formal manager for several people. I’m a manager for the course administrators next to me here in the corridor.*

(Participant no 6)

### Power and identity

Holding a position as an educational leader at the university was perceived as having a lower status than being engaged in research and/or being a research leader. One example of this was the notion that medical education research had a lower status than all other research fields at the university. The vision and mission of the educational agenda at the university was also perceived as being much less defined and unclear than the vision and mission for the research agenda. The status of being an educational leader was however confirmed and authorised mainly by other educational leaders and people interested in education in connection the undergraduate medical programme.

*Education doesn’t generate enough money, it is rather costly. It’s research that counts. To get funded. On the other hand I guess some people have confidence in what I do. I think so. But in hard times…well, its money that matters. So I’d say…well, a weak position at the university.*

(Participant no 13)

*If they don’t show that education really matters at this university then it is never going to be considered as important as other kinds of research and…what should we say…well, what usually is referred to as research. Because educational research at this university still doesn’t count as research. You talk about research and then you talk about education.*

(Participant no 5)

Even though the informants felt as they held a position at the undergraduate medical program within the university, a perceived lack of feedback and dedication to educational issues from an overarching university organisational level contributed to the notion of the position mostly having a symbolic character.

*And there’s no one who once or twice a year sends an email saying “good work” or call and say “good work”. No one. There’s no feedback at all. And the funny thing is that the university talks about how important feedback is. But it’s non-existing on the higher levels. Extremely sad, really.*

(Participant no 16)

Most of the informants had some leadership training before starting the position as an undergraduate medical education leader, but of very different kinds and seldom in connection to the specific task and/or educational leadership. Faculty development offered from a university level was often perceived to be more focused on pedagogical issues than on leadership which caused further notions of the lack of authorisation from the university level.

*But I don’t know if I would call it a leadership programme really. (…) It’s not what you traditionally would call a leadership programme. (…) Yes…Well, it’s… It’s all about educational issues from a leadership perspective, that’s the way I would like to put it. But it’s not a leadership programme.*

(Participant no 13)

## Discussion

Being an educational leader at an undergraduate medical education programme should, according to Gee [2], be defined as an i-identity: an institutional identity stemmed from a specific position at a university. The identity is authorised through the university. However, one of the main prerequisites for this assumption is that the person in the position is actually identifying themselves as a leader. The results from this study show that this is not clearly the case among educational leaders within an undergraduate medical programme.

When taking a closer look at the perceived identity of the educational leaders, “leadership” is in fact not the chosen main identity activity in correlation with their experiences; instead it is “mediation” embedded in a feeling of “ambiguity”. To lead teachers along in change processes is seen as a challenge. Even though research on academic leadership in higher education shows that educational leaders often have difficulties to get other academics/experts to move along in change processes [30–32] there is support for the fact that leaders in undergraduate medical education have an unusually large amount of stakeholders and cultures to take into account in their daily work as reported in the results: different departments, different specialties, different hospital sites, different learning cultures, clinical work and university/education, preclinical and clinical, research and education, hospitals and primary care and also across courses and themes. Constant negotiation between interests and attempts between them to gain ground and win influence over the medical education curriculum is often referred to as “tribalism” [11]; a real and specific activity that seems to have resulted in a strong influence on the educational leaders in undergraduate medical education in this study. Hence, the educational leaders view their own identity on a micro level more as mediators than actual leaders. Hence, one of the baseline criteria of Gee’s theoretical pattern [2] cannot be fulfilled.

Turning away from how the educational leaders perceived their own professional identity and turning towards how they believe to be perceived by others, it is possible to compare and contrast the theoretical and empirical patterns from the results. Even though the educational leaders in this study in fact have a position within an educational programme at a university, the leaders do not feel like their identity is authorised by the university; the university is not granting the rights and responsibilities that go with the position [2]. In Gee’s model [2] this could be explained by the fact that the leaders do not perceive the university to sustain their identity enough; it is rather felt to be ignored. This in turn originates from feelings of lacking interactions and dialogues between the leaders and the university [2] which is mirrored on several levels in the results: education has a lower status than research as well as a perceived lack of feedback and of purposefully designed faculty development. Combined, it all contributes to the feelings of ambiguity towards the educational leadership identity which should be authorised from a university level, but is perceived not to be. The same feeling of ambiguity towards the educational leadership identity is perceived in relationship to the teachers and the difficulties in leading them towards educational development. Further, the results from this study bare a strong resemblance to the literature on identity formation among medical educators. For example, Stenfors-Hayes et al. [7] shows that low status and lack of support, influence and responsibility are all factors involved in the identity formation of medical educators. Hence, there seems to be a parallel process between the identity formation of teachers and the identity formation of leaders within undergraduate medical education: their identity formations takes place in a context which often seems to premier other values than education and development of education.

The results of this study in combination with the literature on identity formation among teachers in medical education [1, 7, 8] also indicates that the supposed shared tasks are not clear and there is no clear mandate from the university level. Further: teachers have difficulties in identifying themselves as educationalists, the educational leaders have difficulties in identifying themselves as leaders and the group lacks a common educational language or joint educational visions. For the educational leaders to get affirmation from the university on their identity, they have to step outside of their roles as educationalists and into their roles as researchers. This in turn indicates a problematic context for potential development of undergraduate medical education as a whole. Finally however, the results also show that even though feedback on the identity as an undergraduate medical education leader is perceived as being scarce from a university level, it is confirmed and hence somewhat authorised on the same organizational level as the leaders themselves; among their own peers in the courses and themes.

## Conclusion

The aim of this study was to contribute to the limited amount of research done on medical education leaders and their identity formation and to specifically shed light on the identity of undergraduate medical education leaders. The study has defined the identity of undergraduate medical education leaders as different compared to Gee’s proposed i-identity [2] which functioned as the blueprint for the pattern matching process in this study. Still, Gee’s power-based identity model and definition of “identity” have been useful when exploring the specific characteristics of the identity of undergraduate medical education leaders; the power perspective has functioned as a lens when zooming in on the specific concept of identity development for educational leaders in undergraduate medical education. Their perceptions of their own professional identity as well as their perceptions of how others view their professional identity provide us with important notions on the complexities on executing their important mission to develop medical education. For example, their perceptions of ambiguity towards the process of trying to lead teachers toward educational development and there is a perceived lack of authorisation of their work from the university level. These are important flaws to observe and correct when improving the context in which they are trying to further develop undergraduate medical programmes.

A practical outcome of the results of this study is first and foremost the facilitation of design of faculty development programs for educational leaders in undergraduate medial education. It is important that universities running medical programmes for students also are running faculty development programs specifically designed for those who are medical education leaders. Not having access to this kind of faculty development may create further feelings of alienation towards the central university level, when medical education leaders are trying to achieve their important mission to further develop medical education. If the goal is to create a functioning community of practice consisting of both teachers and leaders in undergraduate medical education, jointly working towards a high quality medical education programme, it is important that both teachers and leaders have access to qualitative theory-based faculty development programmes designed for their particular needs. The power perspective presented in this study is just one of many applicable theoretical perspectives on educational leadership in undergraduate medical education and therefore also in faculty development programmes for the target group. It is of importance that the challenges of educational leaders’ identity formation exposed in the results of this study also are highlighted and discussed in these programmes to create a better understanding of the context they are operating in as well as of their important mission.

## Additional file

Additional file 1: Interview guide. The interview guide used in the semi-structured interviews with the informants. (DOCX 22 kb)
