# Supplementary material for: May I see your ID, please? An explorative study of the professional identity of undergraduate medical education leaders
Source: BMC Med Educ. 2017 Feb 1;17:29. doi: 10.1186/s12909-017-0860-0 (PMC5286680; doi:10.1186/s12909-017-0860-0)
Supplement: Additional file 1: — Interview guide. The interview guide used in the semi-structured interviews with the informants. (DOCX 22 kb) [file 12909_2017_860_MOESM1_ESM.docx]

Additional file 1

**Interview guide**

**Warm-up question:**

- Could you please describe a typical day at work?

1. **Bibliographical information**

- Name?
- Please describe your academic and professional educational background?
- What other/previous experiences do you have from a leadership position?

1. **Exploring issues of leadership, authority and resistance**

- How do you define leadership?
- Describe your current leadership position
- In relation to leading change in your organisation – what kind of questions and areas can you influence?
- In relation to leading change in your organisation – what types of resistance have you encountered?
- How would you describe your mission?

1. **Introducing change**

- Give an example of an educational change process that has taken place within your context.
- What creates possibilities and/or obstacles in your work towards change and development?

1. **Exploring educational issues**

- How do you perceive your status as an educational leader in your organisation?
- Are there any final issues or comments you would like to add?
